# Genome-Scale Co-Expression Network Comparison across *Escherichia coli* and *Salmonella enterica* Serovar Typhimurium Reveals Significant Conservation at the Regulon Level of Local Regulators Despite Their Dissimilar Lifestyles

**DOI:** 10.1371/journal.pone.0102871

**Published:** 2014-08-07

**Authors:** Peyman Zarrineh, Aminael Sánchez-Rodríguez, Nazanin Hosseinkhan, Zahra Narimani, Kathleen Marchal, Ali Masoudi-Nejad

**Affiliations:** 1 KU Leuven, Department of Microbial and Molecular Systems, Centre of Microbial and Plant Genetics, Leuven, Belgium; 2 Departamento de Ciencias Naturales, Universidad Tecnica Particula de Loja, San Cayetano Alto S/N, Loja, Ecuador; 3 Laboratory of Systems Biology and Bioinformatics (LBB), Institute of Biochemistry and Biophysics, University of Tehran, Tehran, Iran; 4 Ghent University, Department of Information Technology, IMinds, Gent, Belgium; Animal Health and Veterinary Laboratories Agency, United Kingdom

## Abstract

Availability of genome-wide gene expression datasets provides the opportunity to study gene expression across different organisms under a plethora of experimental conditions. In our previous work, we developed an algorithm called COMODO (COnserved MODules across Organisms) that identifies conserved expression modules between two species. In the present study, we expanded COMODO to detect the co-expression conservation across three organisms by adapting the statistics behind it. We applied COMODO to study expression conservation/divergence between *Escherichia coli*, *Salmonella enterica*, and *Bacillus subtilis*. We observed that some parts of the regulatory interaction networks were conserved between *E. coli* and *S. enterica* especially in the regulon of local regulators. However, such conservation was not observed between the regulatory interaction networks of *B. subtilis* and the two other species. We found co-expression conservation on a number of genes involved in quorum sensing, but almost no conservation for genes involved in pathogenicity across *E. coli* and *S. enterica* which could partially explain their different lifestyles. We concluded that despite their different lifestyles, no significant rewiring have occurred at the level of local regulons involved for instance, and notable conservation can be detected in signaling pathways and stress sensing in the phylogenetically close species *S. enterica* and *E. coli*. Moreover, conservation of local regulons seems to depend on the evolutionary time of divergence across species disappearing at larger distances as shown by the comparison with *B. subtilis*. Global regulons follow a different trend and show major rewiring even at the limited evolutionary distance that separates *E. coli* and *S. enterica*.

## Introduction

One of the key issues in system biology is to identify functional orthologous genes. These are genes that not only share sequence ancestry, but also are expected to perform the same function in different organisms. Microarray expression technique is a genome-scale high-throughput experiment which can identify genes with similar function with high accuracy, as genes with similar function tend to have more similar expression profiles.

In a previous study, COMODO was introduced as a methodology which can detect co-expression conservation between two different organisms [1]. COMODO is initialized with finding co-expressed seeds or seed modules obtained from each of the species. These seeds are then gradually expanded in each of the species until a pair of modules is obtained for which the number of shared homologs is statistically optimal relative to the size of the linked modules. The strength of COMODO resides on its ability to automatically prioritize best matching module pairs. The retrieved pairs can cover a large range of co-expression levels (e.g. operon or regulon level conservation) and module sizes.

**Figure 1:**
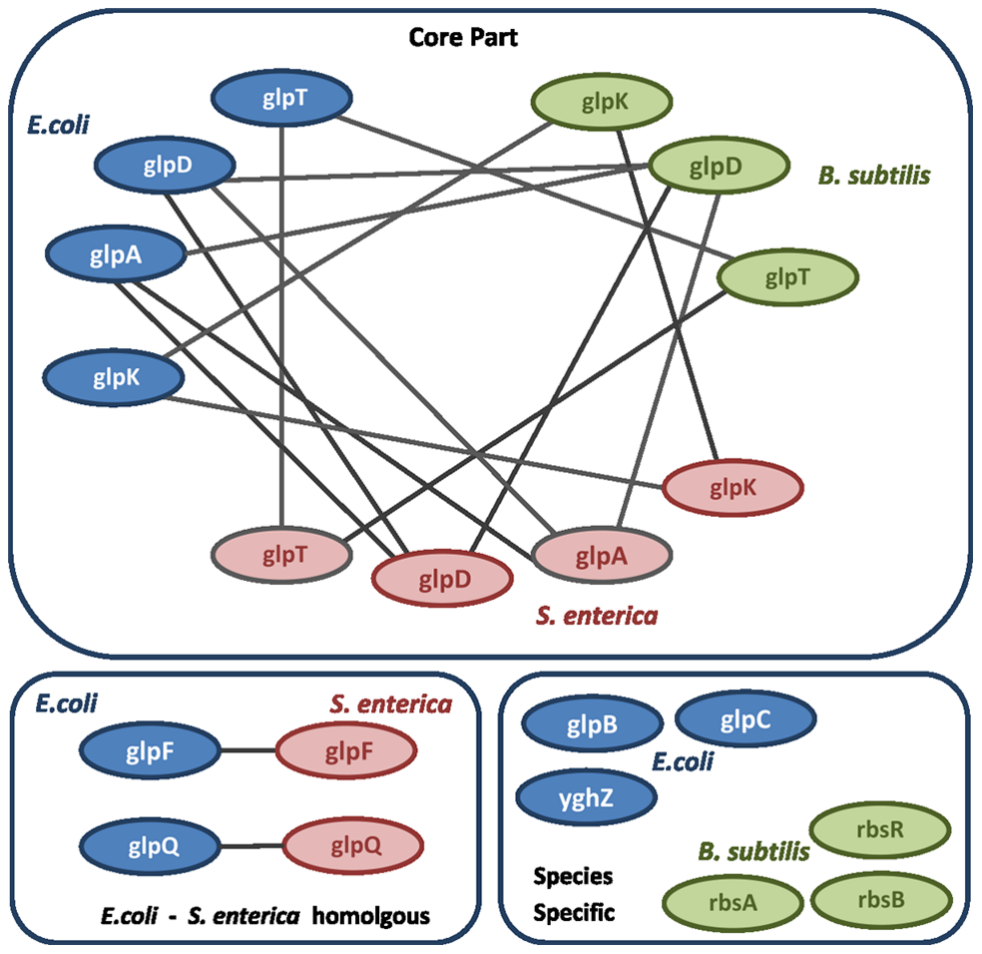
Schematic representation of COMODO output for the first detected module across *E. coli*, *B. subtilis*, and *S. enterica*. Modules in conserved co-expressed triplets are composed of homologous triplets between three organisms (core part). In addition, homologous pairs can be detected which are conserved only between two organisms, that share a mutual co-expression in each of the species. Furthermore, additional genes can also be detected for which the co-expression with the homologous linker genes was found to be species-specific.

In the present paper, we have improved COMODO to detect a larger number of co-expressed seed modules in each organism. In each organism, seed modules are identified by applying a pre-specified maximal stringency threshold (see Materials and Methods section 2.5). We have enabled COMODO to apply a range of pre-specified maximal stringency thresholds to detect more initial seed modules in each organism. In addition, we have extended the optimization criteria to three organisms to detect co-expression conservation across three organisms (see Materials and Methods section 2.4). **Figure 1** presents an example of a detected conserved co-expressed module across three organisms.

Although previous cross-species comparison studies have revealed the conservation of co-expression and regulatory networks between different prokaryotic [1]–[4] or eukaryotic [5]–[9] organisms over diverse ranges of phylogenetic distances, it is still unclear to what extent lifestyle influences the conservation of co-expressed modules and the regulatory network across different phylogenetic distances.

To explore to what extent changes in co-expression conservation relates to organisms' lifestyles, we studied two evolutionary close prokaryotic model organisms: *Escherichia coli* and *Salmonella enterica*. Although these two gram-negative bacteria are evolutionary very close, *S. enterica* is a dangerous pathogen specialized to the host intercellular environment [10]. *S. enterica* can adapt itself to live beside many other habitats within host cells in which it can be colonized. *S. enterica* has therefore the capacity to cope with severe conditions such as low abundance of nutrients and ions thanks to its metabolic versatility e.g. by simultaneously employing several pathways. In order to add an evolutionary perspective to our results and to consider different phylogenetic distances, *Bacillus subtilis* was included as third species in our comparative study of co-expression conservation. Given our species set, we paid a special attention to the genes involved in quorum sensing as the quorum sensing and pathogenesis since these functions may be influenced by lifestyle.

The improved version of COMODO used in the present study to compare expression compendia across three species is available at:

http://bioinformatics.intec.ugent.be/kmarchal/Supplementary_Information_Zarrineh_2010/comodo/

## Materials and Methods

### Microarray compendia

The microarray compendium of *E. coli* was obtained from Lemmens *et al.* [11] and the one of *B. subtilis* from Fadda *et al.* [12]. They contained respectively 870 conditions for *E. coli* and 231 for *B. subtilis*. Microarray compendium of *S. enterica* was obtained from COLOMBOS [13] containing 657 conditions. All three compendia include data from different strains (see **Text S1**, **Table S3**, **Table S4**). The detailed information regarding the strains that were used for microarray experiments, and the impact of using compendia, containing different experimental conditions of various strains has been described in **Text S1** (**Text S1**, **Table S5**, **Table S6**, **Table S7**, and **Table S8**).

### Homology map and sequence similarity

The homology map between different bacteria was derived from the COG database [14], and orthologous gene families were derived using smallest distance approach [15].

### Condition selection for module visualization

For visualization purposes heat maps only display the conditions for which the co-expression behavior was most obvious. Relevant conditions were selected by dividing per condition the mean value of the expression levels in the module by the variance (coefficient of variation). If this coefficient of variation exceeds a predefined threshold (1 in our case), the corresponding condition is visualized.

### Statistics to assess co-expression conservation between two or three organisms

Two data sources, sequence similarity (homology) and gene expression were used to detect genes with conserved expression behavior across multiple organisms. Given the state-of-the-art detecting genes with sequence similarity is straightforward as prediction on direct orthologous gene pairs or homologous gene families across species, are available in several databases [14]–[16]. However, a more challenging task is to combine information on ‘sequence conservation’ or ‘a homology relation’ with co-expression information to automatically search for ‘conserved co-expression modules’ or co-expression modules in each of the species that are linked to each other with a statistically significant number of orthology or homology relations. The main idea behind COMODO is to find proper thresholds to detect co-expressed modules in two or more organisms, in a way that maximizes the observed linked homologous genes using a proper statistical test. COMODO performs in a way that the selected threshold is different for each co-expressed module.

To define proper statistics for the detection of co-expressed modules between two organisms, homology relations can be considered as a bipartite graph (of homologous gene pairs), in which nodes correspond to the genes in each of the organisms and edges represent homology relations between the genes. Given two co-expressed modules, one in each organism, the p-value of observing such linked modules with the homology relations between their composing genes can be calculated by performing a Monte Carlo sampling. To perform Monte Carlo sampling, in each step two edges are shuffled in a way that the distribution of degrees in the bipartite graph remain preserved. This shuffling is carried out by repeatedly selecting at random two edges and crossing them (replacing two homology relations). If two modules Ci and Cj are linked with |T| homology relations, and a shuffling procedure is performed n times, a p-value can be calculated as follow:
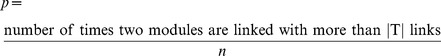



To extend this procedure to three organisms, the homology relations between three organisms can be considered as a tripartite graph (of homologous triplets), in which nodes reflect genes and edges are the homology relations among them. Monte Carlo sampling can be done in a similar way, as explained above, for such tripartite graph. Each homology relation consists of three genes in three organisms linked based on homology. To perform Monte Carlo sampling, in each step two homology relations are chosen, in a way that each two genes of the same organism will be different. Now it is sufficient to reshuffle two links out of three links exists in homology relation. This reshuffling will generate a new tripartite graph which preserves the distribution of out degrees (number of outgoing edges from the nodes) for all nodes in the graph. As an illustration, consider the following homologous triplets: (G11-G21-G31) and (G12-G22-G32), where G stand for gene, the first number in the subindex refers to the species number (i.e. 1^st^, 2^nd^ and 3^rd^ species) and the second number in the subindex refers to the gene id (i.e. gene 1 or gene 2). Genes grouped under the same brackets constitute a homologous gene triplet across three species. We could reshuffle the homology relations from these two homologous triplets as follows:
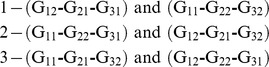



Therefore, if three modules Ci, Cj, and Ck are linked with |T| homology relations and a shuffling procedure is performed n times, a p-value can be calculated as follow:
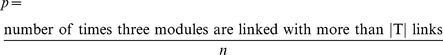



Notice that homology links, connecting only two organisms, are not considered in the formula because only the homology links (gene families) which exist in all three organisms are considered as conserved. These homologous triplets influenced the optimization criteria to optimally define the co-expressed modules (see below).

Running Mont Carlo sampling in each iterative step of COMODO is not computationally feasible because in each step of COMODO Monte Carlo sampling should be performed, and consequently the edge reshuffling procedure should be run for millions of times just for each step. Pearson's chi-square statistic test can be used instead as a proper test to approximate the p-value. The assumption behind the Pearson's chi-square statistic is that the homologous links are evenly distributed in the bipartite (in the two organisms case) and tripartite (in the three organisms case) graphs. Note that nodes with large number of connections do not meet these assumptions and cause problem for estimating the real p-value with Pearson's chi-square statistic test because these connections cause the given homology network skews from an evenly connections distributed network assumed by statistic test. However, genes in our dataset do not have a large number of homology relations with other genes, and most genes only have one or two homologous linking pairs in the other organisms. In addition, the largest degree in our homologous triplet network is 83. These facts justify using Pearson's chi-square statistic to estimate the real p-value.

The corresponding formulation of the Pearson's chi-square statistic was introduced in our previous publication for comparison of co-expression between two species [1]. To formulate the Pearson's chi-square test for detecting co-expression in three organisms, consider *N1* genes in the genome of the first organism, *N2* genes in the genome of the second organism, *N3* genes in the genome of the third organism, and *M* homologous gene triplets derived from the COG database. If we pick three genes randomly, one from each organism, the probability of choosing a homologous gene triplet is equal to 
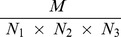
. Therefore, the probability that these genes are not a homologous triplet is 
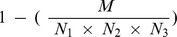
.

Given three modules (one for each organism) containing respectively *g1* genes from the first organism, *g2* genes from the second organism, and *g3* genes from the third organism (where *g1*, *g2*, *g3*<<*N1*, *N2*, *N3* respectively), the expected number of homologous gene triplets that would appear, assuming that the three modules are randomly selected, can be estimated by:
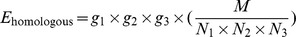



The expected number of non-homologous gene triplets appearing in the module can be estimated by:
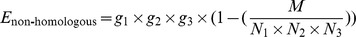



We used the Pearson's chi-square test to assess whether the number of homologous and non-homologous gene triplets in a linked co-expressed module is significantly different from the expected one. A chi-square test with one degree of freedom is as follow:
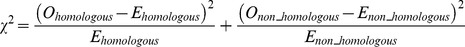



Where *O* and *E* stand for observed and expected values respectively. Note that as the *p*-value might get very close to zero, we use an optimization criterium that maximizes the actual chi-square values instead of minimizing the corresponding *p*-values.

### Application of the methodology to the *E. coli*, *B. subtilis*, and *S. enterica* datasets

The COMODO methodology was expanded to accept a range of prespecified maximal co-expression stringency values to detect more module seeds in each organism. We used five prespecified maximal co-expression stringency values, 0.9, 0.8, 0.7, 0.6, and 0.5, in this study. Similar to the previous publication [1], the Pearson correlation across all conditions was used as the measure for co-expression. In theory, using five prespecified maximal co-expression stringency values results in five different module seeds, but in practice many of these module seeds are identical.

We used COMODO for two organisms to find co-expression conservation between *E. coli* and *S. enterica* with the same setting and filtering procedure as the previous publication [1] experiments.

COMODO was also extended to find expressional conserved modules in three organisms. We applied COMODO to find conserved modules across three bacteria *E. coli*, *B. subtilis*, and *S. enterica*. For three organisms we also used the same settings and filter procedure as in previous publication [1], except for the ‘minimal fraction of homologous versus non-homologous genes’ and the ‘least initial linker genes in each module’ which were set to 0.2. We used 0.2 instead of 0.1 for these two variables in our experiments to reduce the number of linked module triplets in order to make the memory usage more efficient, and also to reach the stopping criteria faster as searching in the best threshold for three modules (each for one organism) can be much slower than two. In addition, the highly conserved co-expressed modules contain much higher ratio of genes linked by homology.

### Annotation of the detected conserved co-expressed modules across *E. coli*, *B. subtilis*, and *S. enterica*

Each conserved co-expressed module detected by COMODO contains a core and a variable component. The core part involves homologous (orthologous) genes for which expression behavior has been conserved across species. The variable component can be the result of either homologous (orthologous) genes truly differently regulated across species, or genes that did not end up in the core part because no significant expression conservation was found with its homologous (orthologous) genes in the other species (spurious results). This latter case could be due to lack of sufficient expression data in the corresponding compendia (as to accurately calculate expression conservation) or noise introduced in preprocessing and processing of expression information to detect co-expression conservation by COMODO.

In order to cope with the amount of ‘noise’ possibly introduced to the variable part of the modules by the aforementioned causes and to do not draw unrealistic conclusions from our results, we performed an enrichment analysis. For enrichment calculation we took into account several sources of gene annotation such us: Gene Ontology (GO) terms, metabolic pathways, and protein complexes of *E. coli* were downloaded from EcoCyc [17]. Metabolic pathways and protein complexes of *B. subtilis* were obtained from BioCyc [18]. Transcriptional interactions were downloaded from RegulonDB [19] and DBTBS [20] for *E. coli* and *B. subtilis* respectively. Enrichment analysis was done based on the hypergeometric distribution corrected for multiple testing by the False Discovery Rate (FDR) [21].

By reporting our results based on enriched categories and because of the statistics behind the enrichment calculation, deviations of the ‘real modules’ by few spurious genes in the variable part will not change the main function(s) assigned to each module e.g. based on enriched GO labels from the biological process domain. For the cases in which we discuss a module in detail (see Results) and derive conclusions about conservation/divergence of expressional behavior across species, we verified that the same annotation categories were enriched in the gene sets corresponding to each species. In two cases (see section 3.2) we manually retrieved the missing genes from the modules by using available operon information and performing a condition selection in the microarray compendia which allowed us to detect significant co-expression of homologous genes across species missed by COMODO.

## Results

### Identifying evolutionary conserved and non-conserved co-expressed modules ACROSS E. coli, B. subtilis, and S. enterica

Applying COMODO to expression compendia of *E. coli* and *S. enterica* resulted in the identification of 211 conserved module pairs that were linked through a statistically significant set of homologous genes (**Table S1**). Applying COMODO to infer modules that were conserved between *E. coli*, *B. subtilis*, and *S. enterica* resulted in 110 conserved module triplets linked through a statistically significant set of homologous genes (**Table S2**).**Table 1** and **Table 2** give an overview of the functional categories in which genes in the conserved co-expressed module across the three organisms are involved. As it can be seen in these tables, the majority of the detected evolutionary conserved modules comprise genes involved in the transport of various substances and pathways of nucleotide, amino acid, carbohydrate, lipid, and co-factor metabolism. Large evolutionary conserved modules (larger in number of genes) in all three species were enriched in ribosomal metabolism and translation, motility and flagella synthesis, and iron acquisition. Two large evolutionary conserved modules related to cellular respiration (anaerobic and aerobic respiration) were detected only in *E. coli* and *S. enterica*. These two large modules seemed to have diverged in the more distant bacterium *B. subtilis*.

**Table 1:** Overview of evolutionary co-expressed conserved modules across three organisms.

Biological process	Module number in Table S2
Nucleobase-containing compound metabolic process	65-66-67-68-75-103-105-106
Amino acid metabolic process	40-41-42-43-44-45-46-47-48-49-71-72-76-102-104
Metabolism of co-factors and vitamins	73-74
Carbohydrate metabolic process	1-2-97-98-107
Transport	10-11-13-14-15-16-17-18-19-20-21-22-23-24-25-26-27-28-29-30-31-32-33-34-35-36-37-38-39-55-76-77-78-79-80-81-82-109
Aerobic respiration	62-63-64-99-100-101
Anaerobic respiration	50-51-52-53
Chaperoning, repair (refolding)	54
Ribosomal metabolism and translation	57-58-**59**-60
Motility and flagella synthesis	**83**-84-85-86-87-88-**89**-90
Iron acquisition	91-92-93-94-95
Cellular response to DNA damage	108
Unknown function	56

The most enriched GO term from the biological process subtree amongst the genes in each module is shown (left column). The numbers of co-expressed modules showing enrichment in the same term are grouped (right column). Conserved co-expressed modules across *E. coli*, *B. subtilis*, and *S. enterica* are their corresponding module numbers as in **Table S2**. The module numbers related to large evolutionary conserved co-expressed module, which contain at least 16 genes in their core part, are highlighted by bold characters.

**Table 2:** Overview of evolutionary co-expressed conserved modules across *E. coli* and *S. enterica*.

Biological process	Module number in Table S1
Nucleobase-containing compound metabolic process	77-78-79-84-86-87-88-93-98-99-100-104-111-127-131-141-142-144-145-171-205-206
Amino acid metabolic process	1-13-14-31-60-61-113-132-133-134-135-136-137-143-152-176-178-179-180-181-200-210
Metabolism of co-factors and vitamins	66-67-140-155-156-157-190-203
Carbohydrate metabolic process	7-9-22-23-24-30-33-63-92-106-107-117-128-146-147-148-149-150-151-197-199-211
Lipid metabolic process	2-8-15-85-92-182
Transport	3-4-5-6-10-12-32-53-55-56-57-95-109-110-125-138-154-167-191
Aerobic respiration	30-39-40-42-155-158-**159**-183-186
Anaerobic respiration	34-35-36-37-41-43-**44**-45-46-47-**48**-49-50-**51**-52-187-188
Chaperoning, repair (refolding)	73-74-75-124
Ribosomal metabolism and translation	68-80-**81**-82-83-89-90-**91**-94-**96**-**97**-153-209
Motility and flagella synthesis	**160**-161-**162**
Iron acquisition and Iron-sulfur metabolism	168-169-170-**172**-173-174-175-208
Cell shape and cell division	72-130-192-207
Response to stress	20-21-70-71-76-112-118-120-193
Response to external stimulus	11-59
Response to chemical stimulus	121
Response to abiotic stimulus	204
Cellular response to DNA damage	202
Signal transduction	25-26-27-126-184-185
Biofilm formation	166
Unknown function	16-17-103-105-115-129-163-164-165

The most enriched GO term from the biological process subtree amongst the genes in each module is shown (left column). The numbers of co-expressed modules showing enrichment in the same term are grouped (right column). Conserved co-expressed modules across *E. coli* and *S. enterica* are their corresponding module numbers as in **Table S1**. The module numbers related to large evolutionary conserved co-expressed module, which contain at least 16 genes in their core part, are highlighted by bold characters.

Cellular respiration was not the only process with conserved expression behavior of the involved genes in *E. coli* and *S. enterica*, and diverged expression behavior in *B. subtilis*. In fact, many smaller modules in size were also detected as conserved only between *E. coli* and *S. enterica*. Some of them were related to signal transduction and response to stimuli regardless of the different lifestyles of these organisms. For example, response to various stimuli were specific to *E. coli* and *S. enterica* like response to stress, response to external, chemical, and abiotic stimulus.

### Regulatory network conservation

Regulatory networks consist of interactions inside the cell that controls gene expression. Interactions between transcription and sigma factors with their targets on the DNA molecule are the well-known parts of regulatory networks in bacteria with large influence on the observed expression. Regulatory interactions could remain preserved or diverged across species during evolution, depending on how species need to adapt themselves to different environments.

In *E. coli* and *B. subtilis*, detected modules were annotated using known regulatory interactions to detect parts of the network that have been conserved or that have diverged during the course of evolution. Unfortunately, the available regulatory information of *S. enterica* is largely incomplete.

**Figure 2:**
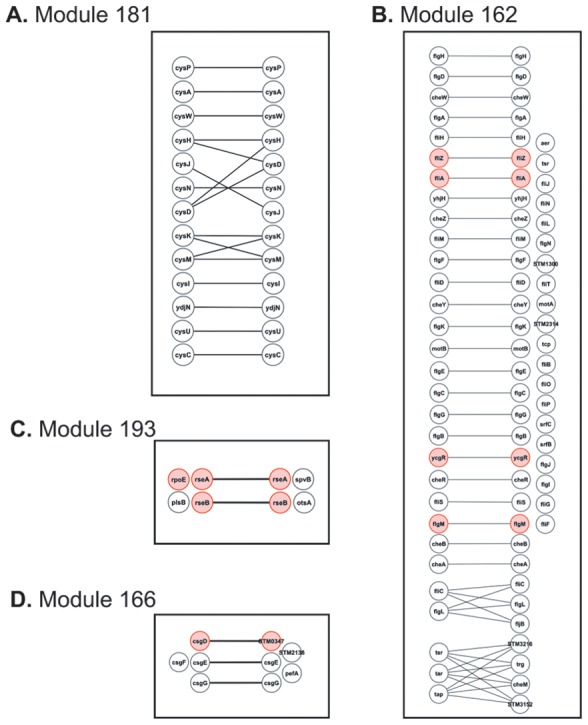
Selected co-expressed conserved modules across *E. coli* and *S. enterica*. **A**. Core part of co-expressed conserved module regulated by transcription factor *CysB* in *E. coli*. Existence of orthologous transcription factors CysB in *S. enterica* makes it highly probable that CysB is responsible for observed co-expression in module 181 of *S. enterica*. In addition, co-expression conservation of ydjN in both organisms may imply that this gene is also a target of CysB, and ydjN is involved in the same biological process as the other genes (cysteine metabolism). **B**. Co-expression conservation of motility and flagerlla synthesis (module 162). Transcription factor FlhCD and sigma factor FliA is known to be responsible for the co-expression of genes involved in this biological process in both organisms. Co-expression conservation of sigma factors FliA and FliZ, anti-sgima factor FlgM, and transcription factor YcgR may also imply the similarity in regulatory interaction conservation. From 20 genes detected as variable part in *S. enterica* just four genes (srfB, srfC, STM1300, STM2314) has not previously been identified as motility and flagerlla synthesis in *E. coli*. The other 16 genes could be detected in *E. coli* if the lower threshold would be used, but using lower threshold could also introduce many new non-linking genes this time in the variable part of *E. coli*. **C**. Co-expression conservation of two anti-sigma factor RseA and RseB in module 193. We expect that sigma factor RpoE is also conserved in co-expression as all these genes are in one operon in both *E. coli* and *S. enterica* (see also **Figure 3B**). **D**. Homologous transcription factors CsgD and STM0347 are co-expressed in linked co-expressed module 166. CsgD also exist in *S. enterica* and probably not detected as co-expressed gene in *S. enterica* because of available condition set in this organism (see also **Figure 3A**).

In **Table 3**, we have highlighted evolutionary conserved transcription factors with conserved co-expressed targets such as Fur, NrdR, LexA, ArgR/AhrC. Co-expression conservation of regulators themselves may also imply the conservation of regulatory interactions across species. In this regard, the detected modules support the co-expression conservation of FliA/SigD, the flagellar sigma factor (sigma 28), and its anti sigma factor, FlgM across all the three bacteria as they co-participate in motility and flagella synthesis (**Table 3**). The sigma factor FliZ, a protein which acts as a sigma(S) inhibitor [22], and the flagellar motility regulator YcgR are also co-expressed but in this case only between *E. coli* and *S. enterica* since these genes do not exist in *B. subtilis* (**Figure 2B**).

**Table 3:** Overview of evolutionary conserved regulators across three organisms.

Regulator	Module number	Targets' co-expression conservation	Regulator co-expression conservation
NrdR	65-66-67-68	Yes	No
Fur	91-92-93-94-95	Yes	No
LexA	108	Yes	Yes
ArgR/AhrC	69-70	Yes	No
FliA/SigD	83-84-85-86-87-88-89-90	Yes	Yes
FlgM	83-89	Yes	Yes
**PurR**	75-105-106	Yes	No
**Mlc/LevR**	10	Yes	No
**GlpR/GlpP**	1-2	Yes	No

Conserved regulators between *E. coli*, *B. subtilis*, and *S. enterica* and the corresponding number of the modules which are enriched as the targets of these regulators. The same module numbers are used as in **Table S2**. **Targets' co-expression conservation**: refers to whether the known targets of the corresponding regulator showed co-expression conservation across the studies species (i.e. they were detected on the core part of the co-expressed module). **Regulator co-expression conservation**: refers to whether the corresponding regulator itself showed co-.expression conservation across the studied species. The non-orthologous regulators between *E. coli* and *B. subtilis* predicted as being functional counterparts i.e. they are responsible for co-expression conserved target genes are highlighted by bold characters.

Given the phylogenetic relations among the three studies species, it can be expected that in the case of the pair of close relatives, *E. coli* and *S. enterica*, most of the orthologous transcription factors are each other's functional counterparts, regulating similar processes. Contrary to this, in the more diverged species pairs i.e. when comparing *E. coli* versus *B. subtilis* or *S. enterica* versus *B. subtilis*, non-orthologous transcription factors may have taken over the regulation of similar processes across species. We actually gathered evidences supporting this hypothesis in the case of PurR, the transcription factor involved in the pruine biosynthesis. The experimentally validated functional counterparts of PurR in *E. coli* and *S. enterica* [23], [24] do not share any sequence homology with PurR in *B. subtilis* and cannot be considered as true orthologs, even though PurR regulon exhibited co-expression conservation in all three organisms in our detected modules. In contrast, the PurR functional counterparts between *E. coli* and *S. enterica* are true orthologous genes exhibiting high sequence similarity.

**Table 4** summarizes a set of orthologous transcription factors in *E. coli* and *S. enterica* which we predicted to be true functional counterparts in these species based on the conserved modules detected by COMODO. As an example, orthologous transcription factors CysB targets are highly conserved in co-expression between *E. coli* and *S. enterica* (**Figure 2A**). In addition, based on the co-expression conservation of ydjN in both organisms, we predicted that this gene is also a target of CysB in these organisms, and ydjN is involved in cysteine metabolism like other target genes of CysB. The majority of the detected conserved transcription factors in **Table 4** are local regulators (such as operon regulators), controlling the expression of few genes. Other examples of transcriptional regulators that only show conservation between *E. coli* and *S. enterica* but not in *B. subtilis* include the self-regulatory transcription factors MtlR, LldR, IscR, NtrC (glnG), PhdR, and Fis (**Table 4**). Two anti-sigma factors RseA and RseB are also conserved in expression only between *E. coli* and *S. enterica* (**Table 4** and **Figure 2C**). Sigma factors and anti-sigma factors are generally known to be highly conserved across evolutionary close bacteria such as *E. coli* and *S. enterica* [25]–[27]. In summary, a large fraction of the known sigma, anti-sigma factors and local regulators (with fewer target genes) seem to be highly conserved between *E. coli* and *S. enterica* (**Table 4**).

**Table 4:** Overview of evolutionary conserved regulators across *E. coli* and *S. enterica*.

Regulator	Module number	Targets' co-expression conservation	Regulator co-expression conservation
TrpR	210	Yes	No
CysB	180-181	Yes	No
NtrC(glnG)	13-110-200	Yes	Yes
ArgR	13-132-133	Yes	No
Fis	84-96	No	Yes
PhdR	96	No	Yes
PurR	99-141-142-144-145-211	Yes	No
PepA	143	Yes	No
LsrR	3-5-6-7-9	Yes	No
GalS	7-10-197	Yes	No
GalR	7-10-197	Yes	No
FadR	7-8	Yes	No
MelR	22	Yes	No
MalT	23-24	Yes	No
SrlR	28	Yes	No
MtlR	63	Yes	Yes
GcvA	211	Yes	No
CsiR	15	Yes	No
AccB	85	Yes	No
PrpR	182	Yes	No
LldR	183	Yes	Yes
IclR	30-42	Yes	No
GlpR	187-188	Yes	No
LexA	202	Yes	Yes
RseA	193	No	Yes
RseB	193	No	Yes
RpoE	193	No	Yes
Fur	168-170-172-174-175	Yes	No
IscR	169-173-190	Yes	Yes
SdiA	130	Yes	No
CueR	203	Yes	No
FliA	160-162	Yes	Yes
FlgM	162	Yes	Yes
FliZ	162	Yes	Yes
YcgR	162	No	Yes
FlhCD	162	Yes	No
CsgD	166	No	Yes

Conserved regulators only between *E. coli* and *S. enterica* and their corresponding number of the modules which are enriched as the targets of these regulators. The same module numbers are used as in **Table S1**. **Targets' co-expression conservation**: refers to whether the known targets of the corresponding regulator showed co-expression conservation across the studies species (i.e. they were detected on the core part of the co-expressed module). **Regulator co-expression conservation**: refers to whether the corresponding regulator itself showed co-.expression conservation across the studied species.

**Figure 3:**
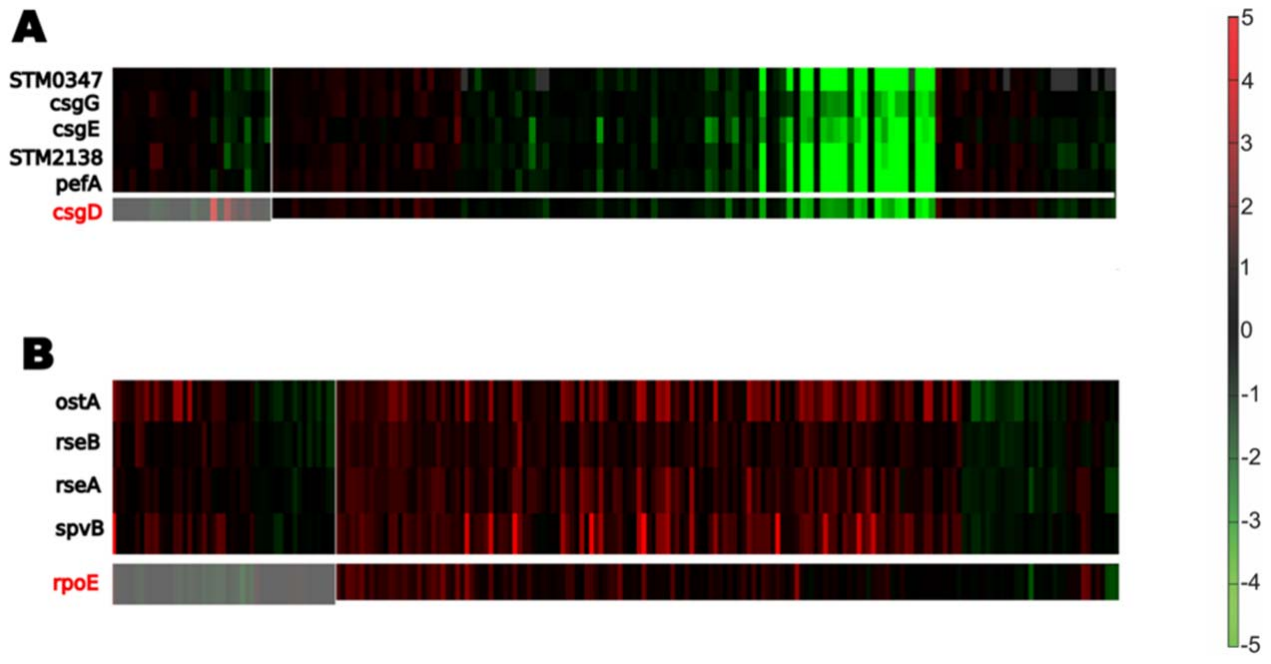
Expression behavior of genes in co-expressed modules 166 (Panel A) and 193 (Panel B) of Table S1 in *S. enterica*. Genes in black are the genes which are found as the co-expressed modules by COMODO. While genes in red (csgD and rpoE) are the ones which are not found in the co-expressed modules, but their ortholgous pair are co-expressed with the *E. coli* counterpart modules. We expect that genes in red (csgD and rpoE) should also be part of their modules as they are in the same operon with some genes of their modules. Shaded areas correspond to conditions not shared for the genes which were not detected as co-expressed in *S. enterica* (red genes). The fact that these conditions are much smaller in number than the conditions genes in red (csgD and rpoE) show co-expression with the rest of the modules genes increases the probability that these genes are actually in those modules.

We also found cases of homologous regulators (not each other's orthologous), which show similar co-expression conservation between *E. coli* and *S. enterica*. This implies the possibility that the homologous regulators are true functional pair. For example, the *S. enterica* transcription factor STM0347 and its *E. coli* homologous pair CsgD appear in the linked co-expressed module 166 (**Figure 2D**, **Figure 3A**, and see also section 3.3). As another example, two co-expressed *E. coli* transcription factors UidR and FeaR show homology and conservation in expression with two co-expressed *S. enterica* transcription factors STM0580 and STM0581 respectively in linked co-expressed module 201, but they are not orthologous pairs. This second example provides more support to the idea that homologous transcription factors, which are not true orthologous transcription factors, may take over the similar function across species.

We also had a look at transcriptional regulators that are known to be highly conserved across the three studied species, but that were not among the detected conserved co-expressed modules by COMODO. The BirA regulator was the only well-known conserved case in three organisms that was not detected by COMODO. In fact, COMODO detected this regulator (and its regulon) as conserved only between *E. coli* and *B. subtilis* in our previous publication [1]. Therefore, we assessed the co-expression of BirA regulon in *S. enterica*. We observed that the average co-expression value among the *S. enterica* regulon of BirA was fairly low (0.106). Unlike *S. enterica*, the average co-expression values of BirA regulon in both *E. coli* and *B. subtilis* were remarkably high (0.45 and 0.766 respectively). In addition, the lowest pairwise co-expression value across the BirA regulon members in *S. enterica* was -0.15, which is much below zero, the value that can be expected by chance. The observed low co-expression among BirA regulon might be partially due to the lack of tested conditions in the *S. enterica* expression compendium. In addition, undetermined noise in the applied conditions which was resulted from different sources such as technological limitations associated with 2-colour microarray experiments and the preprocessing of raw data during the creation of the cross-platform compendia might have played a role. 2-colour microarray experiments, which constituted a large portion of used data source, introduces much higher amount of noise in comparison to more advanced technologies such as RNA-seq. By considering that the co-expression values between gene pairs laid in an interval between [−1 1] and exhibited a normal distribution with mean and variance equal to zero and one respectively, Z-statistics could verify that BirA regulon exhibited co-expression in *S. enterica* (p-value = 1.8×10^−5^). Meanwhile, 56% of genes exhibited average co-expression greater than −0.15 which was the lowest gene pair co-expression of BirA regulon. Therefore, we could presume that the technological limitations and the preprocessing of expression data introduced large amounts of noise which caused two tightly co-expressed genes in one operon to exhibit lower co-expression values than random genes. This could be prevented if the expression compendia had been built by using a more accurate technology such as RNA-seq.Since a comprehensive regulatory interactions database does not exist for *S. enterica*, it is extremely hard to know which other conserved transcription factors across *E. coli* and *S. enterica* were also not detected by COMODO.

### Expression comparison of genes involved in quorum sensing and pathogenicity

We explored the co-expression of genes involved in quorum sensing and pathogenicity comparatively in *S. enterica* and *E. coli*, as these functionalities are directly linked to an organism's lifestyle. Quorum sensing is a mechanism that bacteria use to coordinate their behavior in various environments. Interestingly, from nine gene products known to be responsible in quorum sensing (GO:0009372) in *E. coli* [28], four genes lsrK, lsrG, lsrB (modules 5,6,7) and luxS (module 126) were found conserved in co-expression, and two genes mazF and mqsR does not have orthologous counterparts in *S. enterica*. LuxS was the only gene product, identified as quorum sensing (GO:0009372) in *S. enterica*.

As *S. enterica* is a severe human pathogen, we also looked at gene products related to pathogenesis (GO:0009405). Six *E. coli* genes and 78 *S. enterica* genes were listed as pathogenesis related genes [28], but the majority of these genes were not found among the detected conserved modules. The only two exceptions were lppB (module 126) and flagellar sigma factor fliA (module 160 and 162). In fact, out of 78 gene products known to have a pathogenesis related activity in *S. enterica*, 55 of them do not have an orthologous counterpart in *E. coli*.

Although the large difference in the content of pathogenicity related genes can be seen as the major reason behind the differences in pathogenicity between *E. coli* and *S. enterica*, it is still worth to explore conserved co-expressed modules with pathogenesis related genes in their variable parts. Interesting examples were found in the co-expression conserved modules 17, 18, 19, 20, and 21 across *E. coli* and *S. enterica* (**Table S1**) since it gives insights into the functionalities of other genes, co-expressed with pathogenesis related ones. These modules share a large number of genes in their core parts, and these genes involved in the response to several different stresses. In addition to these stress related genes, two biofilm formation regulators, BssR and GlgS, and one biofilm related gene yjfO were found co-expressed in the variable part of module 17 of *E. coli* and module 20 of *S. enterica*. Three pathogenesis related genes, sseA, slyA, and STM1583 were found in modules 17, 18, 19, and 20 of *S. enterica*. STM1583 is a *S. enterica* specific gene with no orthologous counterpart in *E. coli* that promotes survival in the host environment. sseA and slyA increase resistance to antibiotics and survival in the macrophage environment.

**Figure 4:**
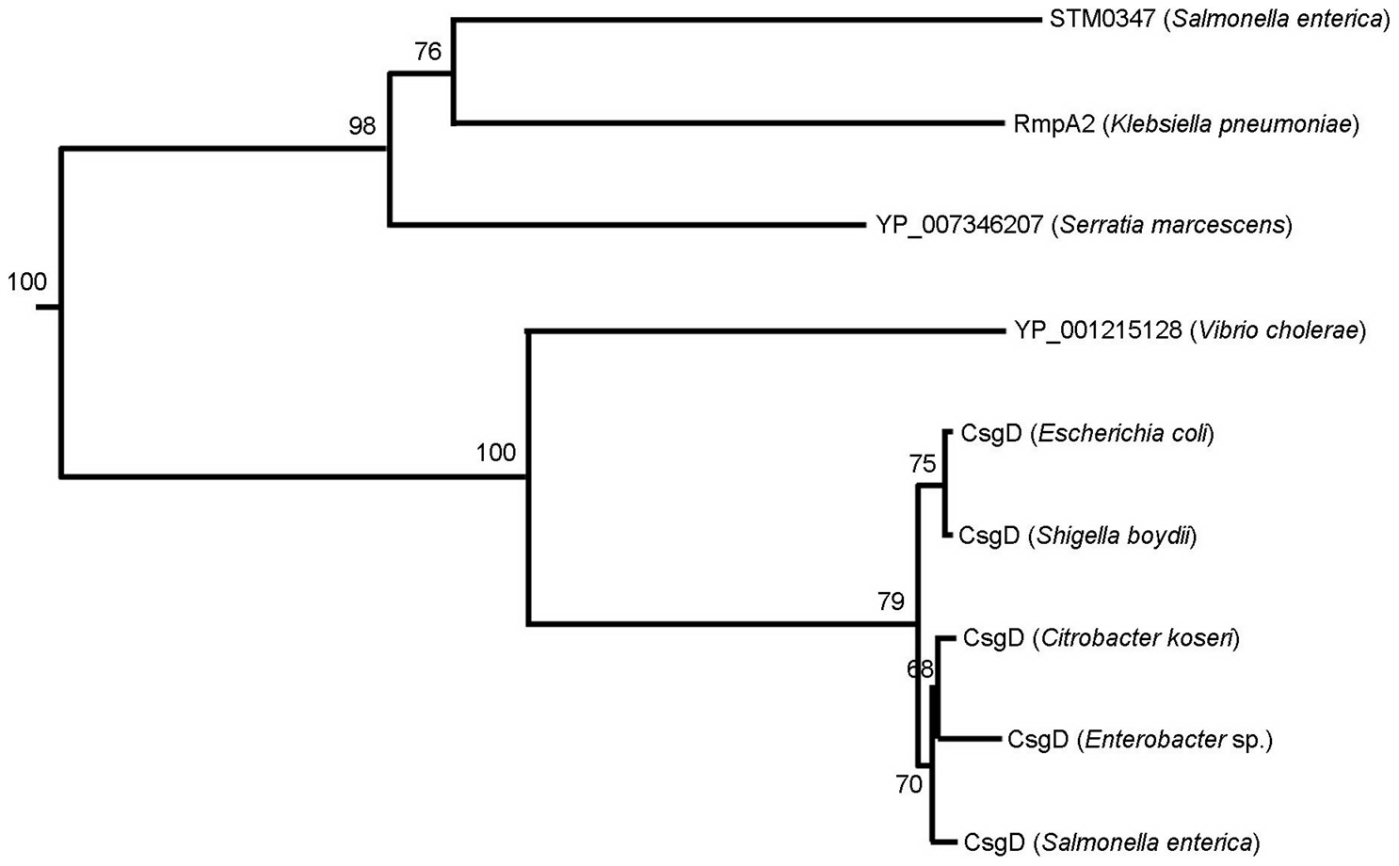
Phylogenetic tree of STM0347 and CsgD. Both proteins were used as queries for BLAST searches to retrieve their closest relatives. Collected sequences were aligned using CLUSTALW [31] and the resulting alignment file used as input for the program ‘neighbor’ of the PHYLIP tree [30] to derive the tree. A total of 100 bootstrap replicates were generated (numbers on the branches). STM0347 and CsgD (*Salmonella enterica*) are far apart on the tree suggesting they have evolved from each other long time ago and might be involved in different functions.

Co-expressed conserved module 166 is another interesting example of a conserved module containing *S. enterica* specific pathogenesis related genes (**Figure 2D** and **Figure 3A**). The core part of this co-expressed conserved module consists of csgDEFG operon, which is involved in the Curli assembly. Curli fibrils are involved in biofilm formation, host colonization, and survival in different environments [29]. Two distinct operons csgBAC and csgDEFG are known to be involved in Curli assembly in both *E. coli* and *S. enterica*. The transcription factor CsgD is known to be the activator of the csgBAC operon but to do not participate in the activation of the genes in the csgDEFG operon [29]. We found the STM0347 protein in the variable part of the co-expressed conserved module 166 which exhibits sequence similarity to CsgD. Despite this sequence similarity (both being LuxR-like proteins), a phylogenetic gene tree of the closest homologs of csgD and STM0347 respectively suggest that these two ‘analogous’ genes are not each others' close relatives and might have different functional roles in *S. enterica*. Still, STM0347 could have been evolved to be a regulator of pathogenesis related genes. The closest non-salmonella homologous gene to STM0347 is RpmA2 (**Figure 4**), which plays role in activation of capsule biosynthesis in *Klebsiella pneumonia*, and RpmA2 has sequence similarity to RcsA protein in *E. coli* which is also a regulator of capsule synthesis [32]. In addition to STM0347, two other genes, pefA and STM2138, were detected in the variable part of *S. enterica* which have no orthologous counterparts in *E. coli*. STM2138 is known to be related to pathogenesis (GO:0009405).

Module 193 in **Table S1**, is another conserved co-expressed module composed of genes related to stress sensing, which contains a pathogenesis related gene, spvB, in the variable part of *S. enterica* (**Figure 2C** and **Figure 3B**). The core part of this conserved co-expressed module consists of genes in conserved rpoE-rseABC operon. RpoE is a sigma factor involved in the response to extracytoplasmic/extreme heat. RseA and RseB, also found in the core part, are two anti-sigma factors. The variable part of the *S. enterica* module includes OstA, which is a protein responsible to osmotic stress.

## Discussion

Co-expression can highlight functional similarity of homologous genes across different species [9]. We could extend COMODO to detect co-expression conservation across three species, and explore the conservation of functions as well as regulatory interactions in varying phylogenetic distances. To this end, we applied the extended COMODO to detect co-expression conservation across *E. coli* and *S. enterica* and *B. subtilis*. We could detect conserved biological processes across these three organisms, as well as biological processes which were only conserved across the closely related species *E. coli* and *S. enterica* such as aerobic and anaerobic respiration. Interestingly, many modules related to response to various stimuli and signal transductions were among the biological processes which were just conserved across the two evolutionary closer species *E. coli* and *S. enterica*, even though some aspects of their lifestyles are remarkably different.

The conservation and divergence of the co-expressed genes illustrate the evolutionary path that each species might go through to adapt itself to the environment, but more importantly the regulatory network responsible for the observed expression should evolve rapidly not only to control the expression of genes involving in different biological processes, but also to enable the organism to interact to convey various signals from environment into the cell. Therefore, the structure of regulatory network is highly divergent even for two closely related organisms regardless of high conservation of observed co-expression [2]. We observed the conservation of few biological processes in all three organisms which is in line with previous knowledge, as the conservation of the target of transcription factors and their upstream binding site motifs have been discussed in depth in separate focused papers for Fur [33], [34], NrdR [35], LexA [36], birA [37], ArgR/AhrC [38]). In addition, we could predict some regulatory network conservation just in *E. coli* and *S. enterica* (and not in *B. subtilis*). The observed conserved regulators were basically local regulators, sigma factors and anti-sigma factors which are known to be highly conserved [22], [25], [39]–[42], and also some self regulatory transcription factors.

The fact, that we observed high co-expression conservation across *E. coli* and *S. enterica*, even conservation in various stimuli and signal transductions, makes it harder to answer the question of what causes their divergence in lifestyles (severe pathogenicity of *S. enterica*). Therefore, we investigated genes involved in quorum sensing and pathogenesis. Four genes out of nine genes involved in quorum sensing in *E. coli* were found to be conserved in co-expression with their counterparts in *S. enterica*. The major source of difference between the two organisms most likely comes from their inventories of genes involved in pathogenesis. Still, we looked at the *S. enterica* conserved co-expressed modules which contained pathogenesis related genes in their variable part. Several modules, with genes related to ‘response to different kinds of stress’ and one module related to ‘Curli assembly’, has contained pathogenesis related genes in the variable part of *S. enterica*. We therefore speculate that the regulation of pathogenesis related genes in *S. enterica* is sort of coupled to those of genes involved in the response to several stresses and ‘Curli assembly’. The latter is supported by the fact that we found a potential regulator (STM0347) of pathogenesis related genes in module 166 which seems to have evolved to be a member family of the regulators of the Curli assembly. In fact, Curli assembly has direct relations with pathogenicity.

Even though we observed high conservation at the level of local regulators, we still expect that a massive regulatory rewiring has been occurred over the regulons of global regulators in *S. enterica* and *E. coli*. We expect this because the conservation of regulons at the both levels of local and global regulators could lead to detection of large conserved co-expression modules by COMODO. The only exception can be respiration, both anaerobic and aerobic, because the detected respiration related conserved co-expressed modules contained large number of genes which cannot be the result of conservation of local regulators alone. This expected massive rewiring of the regulons of global regulators might have enabled *S. enterica* to employ several evolutionary conserved pathways leading to high survival rates in severe conditions within host cells [10]. A recent RNA-seq transcriptomics analysis of 22 distinct infection-relevant environmental conditions has revealed that in average around 63% of genes are expressed in an individual infection-relevant condition [43]. In addition, it has been mentioned that 86% of all *S. enterica* genes are expressed in at least one environmental condition, and it has been concluded that the expression of salmonella genes are highly responsive to environmental perturbations [43]. The fact, that co-expressed modules are highly conserved in phylogenetically close organisms, and we detected significant conservation of co-expression in the regulon of local regulators and sigma factors, makes our argument stronger regarding the occurrence of a massive regulatory rewiring in the regulon of global regulators. This rewiring has enabled *S. enterica* to employ conserved co-expressed modules in a way that it can cope with severe intracellular environment.

In conclusion, we investigated two phylogenetically close species *E. coli* and *S. enterica* with some differences in their lifestyles (severe pathogenicity of *S. enterica*), and we could observe some conservation in responses to various stimuli, transductions of different signals and quorum sensing. Even the comparison of the regulatory networks structure based on the available knowledge show some conservation. This shows that transcriptomics comparison cannot explain complex differences in lifestyles of different species. The comparison must be performed at different level including gene inventories, like the pathogenesis related genes in this study. In addition, comparison at the regulatory network seems to be inevitable because recircuiting in this network (e.g. changes in targets of global regulators) may not affect the content of co-expressed modules, but may enable an organism such as *S. enterica* to be highly responsive to environmental perturbations. With advent of new technologies such as ChIP-Seq the whole-genome reconstruction of *S. enterica*'s regulatory network will become possible which can lead to gain a better insight of this organism's lifestyle. As an example study in another bacterium, the regulatory network of 50 transcription factors (around 26% of predicted transcription factors) of *Mycobacterium tuberculosis* was reconstructed using ChIP-Seq technology [44]. This study could clearly highlight the relation between the regulatory network and adaption to hypoxia in this human pathogen. *In silico* reconstruction of the regulatory network of *S. enterica* is another option. For example, the regulatory network of *S. enterica* was reconstructed by integrating the available regulatory interactions for *E. coli* orthologous transcription factors and the use of structural DNA properties [45]. Recent developments for prediction transcription factor binding sites by combining the sequence similarity and biophysical properties of protein-DNA complexes will lead to more accurate *In silico* regulatory network reconstruction [46]. The learned lessons of this study will be helpful to reconstruct *S. enterica*'s regulatory network in future.

## Supporting Information

Table S1 **Detailed description of the 211 pairs of modules with conserved co-expression behavior between** ***E. coli*** **and** ***S. enetrica*** **.** **Module ID**: ID assigned to the conserved module pair. **Core part**: homologous gene pairs found in core part of the conserved co-expressed modules. orthologous gene pairs are connected by red lines, and non-orthologous ones are connected by black lines. Genes written in red are regulators and genes written in orange are involved in quorum sensing and pathogenecity. ***E. coli*** **variable part**: genes belonging to the variable part of the conserved modules in *E. coli*. Genes written in red are regulators. ***S. enterica*** **variable part**: genes belonging to the variable part of the conserved modules in *S. enterica*. Genes written in orange are involved in quorum sensing and pathogenecity. ***E. coli*** **GO**: GO terms that were found to be enriched in the modules of *E. coli* (core + variable part). ***E. coli*** **KEGG Pathways**: KEGG pathways that were found to be enriched in the modules of *E. coli* (core + variable part). ***E. coli*** **regulators**: Regulators that could be assigned to the modules of *E. coli* (core + variable part). Based on the enrichment of these modules in target genes of previously characterized regulons (as determined by RegulonDB).
(XLS)

Table S2 **Detailed description of the 110 pairs of modules with conserved co-expression behavior between** ***E. coli, B. subtilis*** **, and** ***S. enetrica*** **. Module ID**: ID assigned to the conserved module pair. **Core part**: homologous gene triples found in core part of the conserved coexpressed modules. Orthologous gene triples are connected by red lines, and non-orthologous ones are connected by black lines. Genes written in red are regulators. ***E. coli*** **-** ***B. subtilis*** **homologuos**: homologous gene pairs found conserved just between *E. coli* and *B. subtilis*. Genes written in red are regulators. ***E. coli*** **-** ***S. enterica*** **homologuos**: homologous gene pairs found conserved just between *E. coli* and *S. enterica*. Genes written in red are regulators. ***B. subtilis*** **-** ***S. enterica homologuos***: homologous gene pairs found conserved just between *B. subtilis* and *S. enterica*. Genes written in red are regulators. ***E. coli*** **specific**: genes only belonging to the conserved modules in *E. coli*. Genes written in red are regulators. ***B. subtilis*** **specifc**: genes only belonging to the conserved modules in *B. subtilis*. Genes written in red are regulators. ***S. enterica*** **specific**: genes only belonging to the conserved modules in *S. enterica*. ***E. coli*** **regulators**: Regulators that could be assigned to the modules of *E. coli*. Based on the enrichment of these modules in target genes of previously characterized regulons (as determined by RegulonDB). Conserved regulators in all thress organisms are highlighted in red. ***B. subtilis*** **regulators**: Regulators that could be assigned to the modules of *B. subtilis*. Based on the enrichment of these modules in target genes of previously characterized regulons (as determined by DBTBS). Conserved regulators in all thress organisms are highlighted in red.
(XLS)

Table S3 **The number of compiled conditions related to each strain of S.** ***enterica*** **.** The strain related to each condition was retrieved in S. *enterica* compendium, by checking the conditions in GEO database. Then for each strain, the number of related conditions is highlighted in the second column of this table. If the strain could not be retrieved from GEO database, the more general terms such as ‘*Salmonella enterica*’ or ‘*Salmonella enterica enterica serovar typhimurium*’ were used.
(XLS)

Table S4 **The number of compiled conditions related to each strain of** ***E. coli*** **.** The strain related to each condition was retrieved in *E. coli* compendium, by checking the conditions in GEO database. Then for each strain, the number of related conditions is highlighted in the second column of this table. If the strain could not be retrieved from GEO database, the more general terms such as ‘*Escherichia coli*’ or ‘*Escherichia coli k-12*’ were used.
(XLS)

Table S5 **Detected conserved co-expressed modules across** ***Escherichia coli k-12 substr. MG1655*** **and** ***Salmonella enterica enterica serovar typhimurium LT2*** **by using COMODO.** **Module ID**: ID assigned to the conserved module pair. **Core part**: homologous gene pairs found in core part of the conserved coexpressed modules. ***E. coli*** **variable part**: genes belonging to the variable part of the conserved modules in *E. coli*. ***S. enterica*** **variable part**: genes belonging to the variable part of the conserved modules in *S. enterica*.
(XLS)

Table S6 **Detected conserved co-expressed modules across** ***Escherichia coli k-12 substr. MG1655 and Salmonella enterica enterica serovar typhimurium SL1344*** **by using COMODO.** **Module ID**: ID assigned to the conserved module pair. **Core part**: homologous gene pairs found in core part of the conserved coexpressed modules. ***E. coli*** **variable part**: genes belonging to the variable part of the conserved modules in *E. coli*. ***S. enterica*** **variable part**: genes belonging to the variable part of the conserved modules in *S. enterica*.
(XLS)

Table S7 **Detected conserved co-expressed modules across** ***Escherichia coli k-12 substr. MG1655*** **and** ***Salmonella enterica enterica serovar typhimurium 14028S*** **by using COMODO.** **Module ID**: ID assigned to the conserved module pair. **Core part**: homologous gene pairs found in core part of the conserved coexpressed modules. ***E. coli*** **variable part**: genes belonging to the variable part of the conserved modules in *E. coli*. ***S. enterica*** **variable part**: genes belonging to the variable part of the conserved modules in *S. enterica*.
(XLS)

Table S8 **Comparing the detected co-expressed modules in the strain restricted compendia with the orgional heterogenous compendia.** Each raw of this table consists of conserved co-expressed modules detected by COMODO applied over heterogenous compendia (**Table S1**), *Escherichia coli k-12 substr. MG1655* and *Salmonella enterica enterica serovar typhimurium LT2* (**Table S5**), *Escherichia coli k-12 substr. MG1655 and Salmonella enterica enterica serovar typhimurium SL1344* (**Table S6**), and *Escherichia coli k-12 substr. MG1655* and *Salmonella enterica enterica serovar typhimurium 14028S* (**Table S7**). The co-expressed modules in each row have some genes (at least one gene) in the core part in common with the core part of some other co-expressed modules in that row. This table facilitates the comparison of detected co-expressed in one COMODO experiment and across different COMODO experiments. In fact detected co-expressed modules usually were detected in most of the four experiments. This can show the stability of the results, as the most co-expressed modules detected in heterogenous compendia and strain restricted compendia were in common.
(XLS)

Text S1 **The detailed information regarding the strains that were used for microarray experiments, and the impact of using compendia, containing different experimental conditions of various strains, over detected co-expressed modules.**
(DOC)
