# Supplementary material for: Genome-Scale Co-Expression Network Comparison across Escherichia coli and Salmonella enterica Serovar Typhimurium Reveals Significant Conservation at the Regulon Level of Local Regulators Despite Their Dissimilar Lifestyles
Source: PLoS One. 2014 Aug 7;9(8):e102871. doi: 10.1371/journal.pone.0102871 (PMC4125155; doi:10.1371/journal.pone.0102871)
Supplement: Text S1 — The detailed information regarding the strains that were used for microarray experiments, and the impact of using compendia, containing different experimental conditions of various strains, over detected co-expressed modules. (DOC) [file pone.0102871.s009.doc]

**Supplementary Text 1**

COMODO detected co-expression conservation across *Escherichia coli*, *Salmonella enterica*, and *Bacillus subtilis*. The co-expression conservation experiments were performed based on the idea

that orthologous genes are highly conserved in co-expression for phylogenetically close strains/organisms. Therefore, the prior expectation was large conservation of the co-expression modules across *E. coli* and *S. enterica*. Phylogentically distance organism, *B. subtilis*, was expected to be less conserved in co-expression, and could be considered similar to outgroup in phylogenetic tree.

COMODO detected conserved co-expressed modules across the mentioned organisms as expected. Indeed, large conservation of co-expression modules across *E. coli* and *S. enterica* was detected. Although the results were in line with the expectation, still some concerns exist regarding the compendia. The reason is that the used compendia included data from different strains, and different strains of each organism may contain some differences at the genome content and perhaps regulation. Even though it is not expected that small differences at the genome level could lead to large changes at the content of conserved co-expression modules, still the impact on the output should be assessed.

To assess the impact of using compendia, containing different experimental conditions of various strains, first it is needed to build compendium for each organism, consisting of experiments performed only on a single strain. At the next step, the conserved co-expressed modules must be detected by COMODO, and then the results must be compared. To this end, first the strain related to each condition was retrieved in each compendium, by checking the conditions in GEO database. **Table S3** shows the number of compiled conditions related to each strain of S*. enterica*, and **Table S4** shows the number of compiled conditions related to each strain of *E. coli*. In both tables, if the exact strain could not be retrieved from GEO database, the more general term such as '*Salmonella enterica*' or '*Escherichia coli*' was used. Regarding *B. subtilis*, the exact strains could not be retrieved from GEO database. For these conditions, just '*Bacillus subtilis*' was mentioned in GEO database. Still it is not expected that the differences in *B. subtilis* strains would affect the conserved co-expressed modules because this organism is phylogentically distant from the other two. Therefore, the conserved co-expressed modules across *B. subtilis* and the other two organisms could be expected to be conserved across bacteria phyla.

As mentioned the next step is to detect the conserved co-expressed modules across different strains of *E. coli* and *S. enterica*, and then the results must be compared with the results of heterogeneous compendia. Three *S. enterica* strains, for which large number of conditions were available in **Table S3**, were selected for this analysis. These strains were the main refrence strain *Salmonella enterica enterica serovar typhimurium LT2*, and two other strains, *Salmonella enterica enterica serovar typhimurium SL1344* and *Salmonella enterica enterica serovar typhimurium 14028S*, for which 99, 42, and 305 expression conditions were available respectively. Considering **Table S4**, it can be concluded that the majority of microarray experiments were performed over *Escherichia coli k-12 substr. MG1655*. Therefore, 154 expression conditions of this strain were used for detecting co-expression conservation.

Finally, COMODO was used to detect co-expression conservation across different selected strains. Applying COMODO over *Escherichia coli k-12 substr. MG1655* and *Salmonella enterica enterica serovar typhimurium LT2* resulted in 190 co-expressed modules (**Table S5**). Applying COMODO over *Escherichia coli k-12 substr. MG1655* *and Salmonella enterica enterica serovar typhimurium SL1344* resulted in 76 co-expressed modules (**Table S6**). Here, the low number of available experimental conditions for SL1344 strain is probably the reason of low number of detected modules. Finally, Applying COMODO over *Escherichia coli k-12 substr. MG1655* and *Salmonella enterica enterica serovar typhimurium 14028S* resulted in 246 co-expressed modules (**Table S7**). Related conserved co-expressed modules are listed in **Table S8**, to compare the detected co-expressed modules in the strain restriced compendia with the orgional heterogenous compendia. **Table S8** can show the stability of the results as most modules detected in heterogenous compendia and strain restricted compendia were in common. Especially large co-expression modules such as ribosomal metabolism, flagellar motility, iron acquision, and respiration were detected even in restricted compendia experiments. On the other hand, the modules, only detected in one experiment, were either small operon-level conserved co-expressed modules, or they were modules with lower accuracy which contained large variable part in comparison to the core part. When COMODO was applied over the heterogenous compendia, it could detect more of the operon-level conserved modules. On the countrary, the modules which were only detected in restricted compendia experiments in many cases consist of large variable part. When COMODO detects few genes in core part, and large number of genes in variable part, it is possible that the detected modules are not true conserved co-expressed modules because of the artifact introduced by using insufficient expression conditions to build microarray compendia.

Based on these experiments, we can conclude that using the compendia which include data from different strains will not highly affect the result of COMODO experiments. The major conserved co-expressed modules across different strains of *E. coli* and *S. enterica* are highly stable. On the other hand, reducing compendia to strain-specific ones is not a good strategy because reducing the number of microarray experimental conditions can lead to obtaining more noisy results.
